# Increased vitamin D receptor expression from macrophages after stimulation with *M. tuberculosis* among persons who have recovered from extrapulmonary tuberculosis

**DOI:** 10.1186/s12879-019-3958-7

**Published:** 2019-04-30

**Authors:** Christina T. Fiske, Amondrea Blackman, Fernanda Maruri, Peter F. Rebeiro, Moises Huaman, Jamie Kator, Holly M. Scott Algood, Timothy R. Sterling

**Affiliations:** 1 Division of Infectious Diseases, Department of Medicine, Vanderbilt University Medical Center, A2209 Medical Center North, 1161 21st Avenue South, Nashville, TN 37232 USA; 2 Vanderbilt Tuberculosis Center, Vanderbilt University Medical Center, A2209 Medical Center North, 1161 21st Avenue South, Nashville, TN 37232 USA; 3 Division of Epidemiology, Department of Medicine, Vanderbilt University Medical Center, Nashville, TN USA; 4 Department of Internal Medicine, University of Cincinnati, Cincinnati, OH USA; 5 University of Maryland School of Medicine, Baltimore, MD USA; 6 Department of Veteran’s Affairs, Tennessee Valley Health Systems, Nashville, TN USA

**Keywords:** *M. Tuberculosis*, Extrapulmonary tuberculosis, Toll-like receptor 2, Vitamin D receptor, Interleukin-1 beta

## Abstract

**Background:**

Independent of HIV infection, extrapulmonary TB (EPTB) risk is increased in women, persons of black race or foreign birth, and by genetic variants in vitamin D receptor (VDR), interleukin-1 beta (IL-1β), and toll-like receptor (TLR)-2; functional correlates are unclear. We evaluated macrophage expression of VDR, TLR2, cathelicidin, and TNF-α, and production of IL-1β in HIV-seronegative persons with previous EPTB, previous pulmonary TB, latent *M. tuberculosis* infection, and uninfected TB contacts. Persons with previous pleural TB were excluded due to enhanced immune responses at the site of disease.

**Methods:**

Macrophages were stimulated with TLR-2 agonist *M. tuberculosis* lipoprotein (LpqH), live and gamma-irradiated *M. tuberculosis*.

**Results:**

*M. tuberculosis* – infected macrophages from persons with previous EPTB had increased VDR expression (29.17 relative value unit increase in median expression vs. uninfected contacts, after adjusting for foreign-born status; *P* = 0.02). Macrophages from persons with previous EPTB had a 38.88 μg/mL increase in median IL-1β production after stimulation with LpqH compared to uninfected contacts (*P* = 0.01); the effect was similar (44.99 μg/mL) but not statistically significant after controlling for foreign-born status. Median 25-hydroxyvitamin D levels were low but not significantly different between groups.

**Conclusions:**

There was increased macrophage expression of VDR after stimulation with live *M. tuberculosis* in persons with previous extrapulmonary TB. If post-treatment VDR expression reflects expression prior to disease, it may identify persons at risk for extrapulmonary TB.

**Electronic supplementary material:**

The online version of this article (10.1186/s12879-019-3958-7) contains supplementary material, which is available to authorized users.

## Background

The World Health Organization has estimated that approximately 10.4 million people were diagnosed with tuberculosis (TB) worldwide in 2016 and 1.7 million persons died [1]. TB, however, presents a conundrum: while one-quarter to one-third of the world’s population is infected with *M. tuberculosis*, [2, 3] only 5–10% of those infected develop active disease [4–6]. This indicates that factors other than the mycobacterium play an important role in TB pathogenesis. Our understanding of the contribution of protective immunity in human TB is incomplete. Innate and adaptive immunity are closely connected in the human immune response to *M. tuberculosis*. Activation of the response involves an interplay of cellular components such as macrophages, dendritic cells, and T cells that release and respond to a plethora of cytokines and chemokines, most notably interleukin (IL)-1β, IL-6, IL-8, IL-12, tumor necrosis factor (TNF)-α, and interferon (IFN)-γ [7]. Hematogenous dissemination likely occurs in all persons infected with *M. tuberculosis*, but subsequent extrapulmonary TB disease does not. Most persons infected with *M. tuberculosis* will develop latent, asymptomatic infection. Identifying factors that facilitate reactivation of infection, extrapulmonary dissemination, and active disease will advance TB prevention efforts by focusing on the small minority of persons at risk of developing active TB, as well as protective immune responses that could be augmented by TB vaccines.

Our group has previously noted innate and adaptive immunologic defects in HIV-seronegative adults with previous extrapulmonary TB compared to persons with previous pulmonary TB and latent *M. tuberculosis* infection (LTBI) [8–11]. In particular, we found that HIV-seronegative persons with previous extrapulmonary TB had lower CD4+ lymphocyte counts and their macrophages produced significantly less cytokines such as IFN-γ, TNF-α, and IL-6 both at rest and after stimulation with *M. tuberculosis.* Additionally, previous epidemiologic work has suggested that female sex, black race, and foreign birth are associated with an increased risk of extrapulmonary TB, independent of HIV infection [12, 13]. We and others have also shown that extrapulmonary TB is associated with genetic polymorphisms in the vitamin D receptor (VDR), IL-1β, and toll-like receptor (TLR)-2 [14, 15]. The current study was undertaken to further evaluate the innate immune response to *M. tuberculosis*, specifically the expression of VDR on macrophages and the production of IL-1β by macrophages in in vitro experimental conditions that simulate in vivo infection with *M. tuberculosis.* Differential expression of VDR and IL-1β could provide new insights into TB pathogenesis and help identify those persons at increased risk of progression from latent *M. tuberculosis* infection to active TB disease.

## Methods

### Study design

We performed a case-control study. Cases were defined as persons with previously treated extrapulmonary TB. We defined three sets of controls: 1) persons with previously treated pulmonary TB, 2) persons with LTBI, and 3) persons who had been exposed to culture-positive pulmonary TB but were not infected (i.e., tuberculin skin test (TST) < 5 mm or negative interferon gamma release assay (IGRA)). Persons with both pulmonary and extrapulmonary TB were counted as extrapulmonary for the purposes of analysis. Inclusion criteria consisted of: age ≥ 18 years at time of diagnosis of TB disease or infection; HIV-seronegative; culture-confirmed disease and either near completion (within one month) or after completion of therapy (for extrapulmonary TB cases and pulmonary TB controls)**,** and TST induration ≥5 mm or positive IGRA assay (for LTBI controls). We did not require persons to complete therapy for LTBI to be enrolled. Only contacts of culture-positive pulmonary TB cases were included as controls—both those with and without evidence of *M. tuberculosis* infection. Contacts of culture-positive pulmonary TB cases were tested for LTBI at the beginning of the contact investigation and 8–12 weeks later if the initial test was negative [16]. Exclusion criteria consisted of: serum creatinine > 2 mg/dL; use of corticosteroids or other immunosuppressive agents at the time of diagnosis or study entry; malignancy; and diabetes mellitus. HIV-positive persons were excluded because of the known increased risk of extrapulmonary TB associated with HIV/AIDS [17–19]. Although it is unclear if local cell-mediated immune responses affect the systemic immune response, we excluded individuals with pleural TB from our study because of their exaggerated immune response at the site of disease, which may differ from other forms of extrapulmonary TB [20].

All participants were enrolled from Tennessee. Extrapulmonary TB cases and pulmonary TB controls were identified from the Tennessee Department of Health TB registry. Ongoing contact investigations at local and regional TB clinics were reviewed to identify patients in the remaining control groups. Demographic and clinical characteristics were collected from the patient or the Tennessee TB registry. The institutional review boards of Vanderbilt University Medical Center, Nashville Davidson Metro Public Health Department, and the Tennessee Department of Health approved the study. Study participants provided written informed consent.

### Sample processing

Each subject had HIV serology, complete blood count, and vitamin D measurement performed. We measured 25-hydroxyvitamin D, the major circulating form of vitamin D. Peripheral blood mononuclear cells (PBMCs) were isolated within 24 h under sterile conditions by Ficoll-Paque (GE Healthcare Bio-Science) density centrifugation. Viability was estimated by trypan blue dye exclusion. PBMCs were divided and two aliquots of 2 × 10^6^ cells/mL were plated in a 12-well flat bottom cell culture plate in antibiotic free RPMI 1640 containing 10% heat-inactivated human serum. Gamma-irradiated H37Rv (10 μl of 1 mg/mL, γ-H37Rv) was added to one well. The plate was incubated for three days in 5% CO_2_ at 37 °C.

### Cell separation and magnetic labeling

Monocytes were isolated from PBMCs using the autoMACS system (Miltenyi, San Diego, CA). CD14^+^ cells were isolated using positive selection with CD14 microbeads (Miltenyi Biotech) and incubated in RPMI 1640 for two hours. After two hours, the cells were washed and re-plated in RPMI 1640 containing 10% heat-inactivated human serum and M-CSF (4 ng/μL; Sigma Aldrich, St. Louis, MO) and incubated for three days.

### Infection with M. tuberculosis

*M. tuberculosis* strain H37Rv (ATCC 27294) was grown to log-phase in an upright, vented tissue culture flask in 7H9 broth supplemented with OADC and 0.05% Tween-80. On day four, macrophages from each experiment were stimulated with the following: a specific TLR2 agonist, 19 kDa LpqH (EMC Micro-collections, 10 μg/mL) [21], γ-H37Rv (10 μg/mL), or live H37Rv (multiplicity of infection of 5–10). One well of macrophages was not stimulated. After a two-hour stimulation and incubation in 5% CO_2_ at 37 °C, media was removed and replaced with media supplemented with 1,25α vitamin D [22]. After overnight incubation, the supernatant was removed and frozen at − 80 °C for ELISA. 500 μl of Trizol was added to each well and macrophages were lysed by pipetting. RNA was extracted using the Qiagen RNeasy Kit (Qiagen, Valencia, CA). Similar procedures were used to isolate RNA after 24 and 48 h.

### ELISA for IL-1β

After 24 and 48 h of incubation, supernatant from separate wells was filtered by centrifugation and frozen at − 80 °C. Supernatants from the 12, 24, and 48 h time points were thawed, diluted 1:10 in assay buffer, and evaluated for IL-1β using the IL-1β Human Antibody Pair kit (Life Technologies, Grand Island, NY).

### M. Tuberculosis killing assay

On day four, PBMCs previously incubated with or without γ-H37Rv were washed in 15 mL 1X HBSS and re-suspended in 1 mL of complete RPMI 1640 and macrophages infected with H37Rv were added. Additionally, there were two wells that contained only infected macrophages. Plates were incubated for three days. On the third day, media was removed from the wells and cells were lysed with 50 μl of a 1:5 dilution of 25% sodium dodecyl sulfate and incubated 10 min at 4 °C. Immediately after incubation, 50 μl 10% bovine serum albumin was added to each tube and mixed. Ten-fold serial dilutions were prepared in 7H9 broth to 10^6^ and spread on pre-warmed 7H10 agar plates, and incubated at 37 °C for three weeks. Colonies were counted and CFU calculated at 14 and 21 days.

### Real-time PCR

Total RNA was extracted from macrophages using TRIzol and subsequently purified using the Qiagen RNeasy Mini Kit. cDNA was synthesized using the iScript cDNA Synthesis Kit (Bio-Rad, Hercules, CA) according to manufacturer’s instructions. For qRT-PCR, we used the relative gene expression method (Relative Unites = 2^-ΔΔCt^). Macrophage expression levels of VDR, cathelicidin (CATH), toll-like receptor 2 (TLR2), and tumor necrosis factor-alpha (TNF-α) were measured by quantitative PCR (qPCR) using gene-specific TaqMan Gene Expression Assays (Applied Biosystems, Foster City, CA) on the StepOne Real-Time PCR instrument (Applied Biosystems). GAPDH was used as a normalizer. Specific primers’ GenBank accession number and assay ID are provided in Additional file 1: Table S1).

### Sample size

Based on log-transformed data from a study that evaluated TLR2 mRNA expression after stimulation with *M. tuberculosis* H37rV in active pulmonary TB patients, [23] we estimated a sample size of 12 cases and 24 in each control group would provide 80% power at 5% two sided-significance level to detect a 1.0 log difference. Based on natural log transformed data from a study evaluating TLR2 surface expression by flow cytometry in patients with active TB, [24] we estimated a sample size of 9 cases and 18 persons in each control group would provide 80% power at 5% two sided-significance level to detect a 2.0 log difference. A sample size of 15 extrapulmonary TB cases and 30 persons in each of the 3 control groups was estimated to provide 80% power to detect a 2.5 fold difference in VDR mRNA and a 2-fold difference in cathelicidin mRNA between cases and latent infection controls.

### Statistical analysis

Differences in demographic and clinical factors across exposure categories were assessed using non-parametric tests: Fisher’s exact test for differences in proportions for categorical variables, and the Kruskall-Wallis test for differences in distributions of continuous variables. Experiments were performed in duplicate. Gene expression outcome was a continuous measure (in relative value units; RVU), as was IL-1β production (in μg/mL) and accordingly, the non-parametric distribution of each outcome was described using medians and inter-quartile ranges. Unadjusted and adjusted differences in median values of gene expression and IL-1β by exposure group were determined by quantile regression, and standard errors were calculated via bootstrapping with 20 replicates [25–27]. Because three markers were compared simultaneously in each regression model, the Holm-Bonferroni correction was applied to control the family-wise error rate across multiple tests [28, 29]. Due to a limited sample size for analysis, we could accommodate only a small number of covariates in our models. Because foreign-born status differed by patient group (Table 1) and outcome, we controlled for foreign-born status in the adjusted model of the outcome biomarkers. We did not control for sex or race because the outcome biomarkers did not vary by sex or race. All tests were two-tailed, and estimates were statistically significant if the corrected *p* < 0.00555. All statistical analyses were performed using Stata 12.1 (Stata Corporation, College Station, TX).

**Table 1:** Clinical and demographic characteristics of the study population

Clinical Characteristics	Extrapulmonary (*N* = 12)	Pulmonary TB (*N* = 20)	Latent TB Infection (N = 20)	Uninfected (*N* = 21)	*P*-value
Age in years	41 (31, 61)	58 (44, 66)	44 (32, 54)	49 (32, 53)	0.06
Female sex (%)	5 (42)	6 (30)	15 (75)	12 (57)	0.03
Black race (%)	3 (25)	3 (15)	12 (60)	8 (38)	< 0.01
Hispanic ethnicity (%)	3 (25)	2 (10)	3 (15)	0 (0)	0.10
Foreign born (%)	8 (67)	2 (10)	4 (20)	1 (5)	< 0.01
Alcohol use (4 or more times/week)	3 (25)	12 (60)	7 (35)	11 (52)	0.19
Tobacco use (≥10 cigarettes/day)	2 (17)	12 (60)	5 (25)	2 (10)	< 0.01
Months from treatment completion to blood draw	30.2 (10.7, 68.7)	22.3 (8.7, 32.4)	12 (12, 12)^a^	N/A	0.35
WBC (per mm^3^)	7.4 (5.5, 8.4)	7.1 (5.4, 9.8)	6.7 (5.7, 8.8)	7.8 (5.9, 9.6)	0.80
Monocytes (per mm^3^)	6.5 (6.0, 8.5)	6.0 (5.0, 8.0)	6.0 (5.6, 7.0)	6.0 (5.0, 7.6)	0.79
CD4^+^ lymphocytes (per mm^3^)	1021 (713, 1263)	994 (573, 1135)	1152 (990, 1447)	1285 (948, 1432)	0.08
CD8^+^ lymphocytes (per mm^3^)	562 (375, 647)	583 (311, 761)	622 (461, 856)	540 (362, 635)	0.68

Values are presented as number (%) for categorical variables and median (inter-quartile range) for continuous variables

*P*-values are from the Fisher’s Exact test for differences in proportions for categorical variables, and the Kruskall-Wallis test for differences in the distribution of continuous variables

^a^Three participants with LTBI were documented to have completed treatment

## Results

We enrolled 12 persons with previous extrapulmonary TB, 20 with previous pulmonary TB, 20 close pulmonary TB contacts with LTBI, and 21 close pulmonary TB contacts with a negative TST and/or IGRA. All participants with previous TB disease had completed anti-tuberculosis treatment. We had limited information on treatment of persons with LTBI. One person with LTBI completed nine months of isoniazid, two subjects completed 3 and 5 months of treatment, respectively, and four subjects started treatment but did not complete it. The demographic and baseline characteristics of the study population are shown in Table 1. There was a lower percentage of women among persons with previous pulmonary TB compared to the other study groups. Persons with previous pulmonary TB also were more likely to smoke tobacco compared to the other groups. Persons with previous extrapulmonary TB were more likely to be born in countries other than the United States. Sites of disease among extrapulmonary cases included lymphatic (*n* = 4), vertebral (*n* = 2), laryngeal (*n* = 1), meningeal (n = 1), miliary (n = 1), genitourinary (n = 1), and bone/joint (n = 2). Six persons had both pulmonary and extrapulmonary TB.

**Fig. 1:**
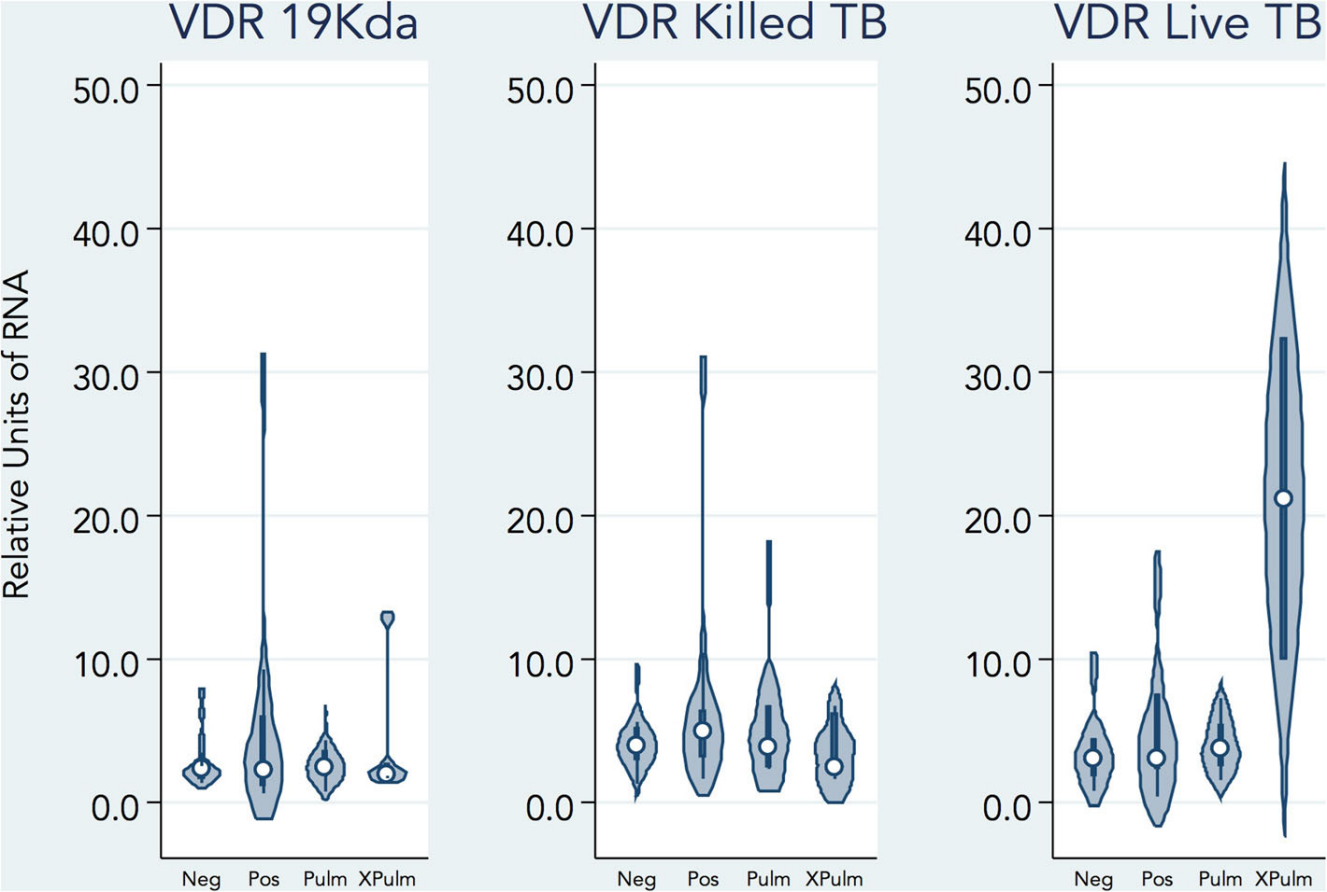
Violin plot showing medians and distributions of *VDR* expression (relative units) by patient group and macrophage stimulation condition. Expression of vitamin D receptor (*VDR)* in PBMC derived macrophages from uninfected persons, latently infected persons and persons with previous pulmonary TB and previous extrapulmonary TB after overnight stimulation. Macrophages were stimulated for two hours with live *M. tuberculosis* (H37Rv, MOI 5–10), gamma-irradiated *M. tuberculosis* (10 μg/mL), and *M. tuberculosis* lipoprotein (LpqH, 10 μg/mL). *VDR* expression levels were assessed by qPCR and normalized to *Gapdh* levels. Experiments were performed in duplicate

To investigate the impact of stimulation on macrophage gene expression, real time PCR was performed. Macrophages were stimulated with LpqH, live *M. tuberculosis,* or gamma-irradiated *M. tuberculosis.* After overnight stimulation of macrophages with live *M. tuberculosis*, VDR expression was markedly increased in participants with previous extrapulmonary TB compared to persons with previous pulmonary TB, LTBI, and uninfected TB contacts (Table 2 and Fig. 1). This difference remained after controlling for foreign-born status, (*p* = 0.02) but not after adjusting for multiple comparisons. In comparison, there was no significant difference between study groups when macrophage VDR expression was measured after overnight stimulation with 19 kDa LpqH (a specific TLR2) agonist and γ-irradiated *M. tuberculosis*. There was no significant difference between the four groups in expression of TLR2, cathelicidin, or TNF-α in unstimulated or stimulated conditions (data not shown).

**Table 2:** Expression of vitamin D receptor (VDR) on macrophages after two hours of macrophage stimulation

Factor-by-Group	Vitamin D receptor expression (in relative value units; RVU)					
	Unadjusted difference in median expression			Adjusted difference in median expression^a^		
	β	95% CI	*P*-value	β	95% CI	*P*-value
LpqH						
Uninfected	Ref.	Ref.		Ref.	Ref.	
Latent TB infection	−0.08	(−2.14, 1.99)	0.94	−0.15	(−2.35, 2.05)	0.89
Pulmonary TB	0.20	(− 0.42, 0.83)	0.52	0.20	(−0.72, 1.13)	0.66
Extrapulmonary TB	−0.43	(−5.34, 4.49)	0.86	−0.63	(−8.29, 7.04)	0.87
Gamma-irradiated *M. tuberculosis*						
Uninfected	Ref.	Ref.		Ref.	Ref.	
Latent TB infection	0.96	(−1.33, 3.26)	0.40	0.48	(−1.79, 2.75)	0.67
Pulmonary TB	0.48	(−2.61, 3.57)	0.76	0.52	(− 2.15, 3.18)	0.70
Extrapulmonary TB	−1.50	(−5.70, 2.71)	0.48	−1.46	(−6.41, 3.49)	0.56
*M. tuberculosis*						
Uninfected	Ref.	Ref.		Ref.	Ref.	
Latent TB Infection	−0.04	(−6.44, 6.36)	0.99	−0.09	(−6.47, 6.30)	0.98
Pulmonary TB	0.93	(−1.04, 2.90)	0.34	0.93	(−1.66, 3.52)	0.47
Extrapulmonary TB	29.22	(2.36, 56.07)	0.03**	29.17	(5.73, 52.61)	0.02**

Unadjusted and adjusted quantile regression analysis of the four study groups according to stimulus used: LpqH, gamma-irradiated *M. tuberculosis*, and live *M. tuberculosis*

LpqH: 19kda lipoprotein of *M. tuberculosis;* Ref.: reference group

Family-wise error rate controlled for multiple comparisons using the Holm-Bonferroni method

^a^Adjusted for foreign-born status. Holm-corrected *P*-values significant if < 0.0056; **Significant if using *P*-values uncorrected for multiple comparisons (*p* < 0.05)

Prior studies have suggested that low levels of vitamin D may predispose to TB. [30] We found in a previous study that persons with treated TB had lower levels of 25-hydroxvitamin D compared to uninfected controls. [31] In our current study we found that persons with previous extrapulmonary TB had low but similar median serum levels of 25-hydroxyvitamin D (24.4 ng/mL; IQR 15.4, 39.5) compared to persons with previous pulmonary TB (19.9 ng/mL; IQR 16.4, 34.3), LTBI (22.9 ng/mL; IQR 13.8, 25.8), and uninfected TB contacts (18 ng/mL; IQR 10.9, 26.3) (Kruskal-Wallis *p* = 0.23). Vitamin D levels < 30 ng/mL were considered low according to standard commercial breakpoints (LabCorp).

Previous studies have found that TLR2 induction of IL-1β production is important for downstream anti-mycobacterial activity [32], therefore the expression of IL-1β was measured by ELISA. We found that macrophages from persons with previous extrapulmonary TB produced significantly more IL-1β twenty-four hours after stimulation with the 19 kDa LpqH compared to uninfected persons (Table 3). The magnitude of this effect was similar after controlling for foreign birth, but it was not statistically significant. Persons with previous pulmonary TB had increased IL-1β production twenty-four hours after stimulation with live *M. tuberculosis* but these results did not reach statistical significance (*p* = 0.07); results were similar in the analysis that adjusted for foreign birth. In contrast, we found that participants with LTBI had decreased IL-1β production twenty-four hours after macrophage stimulation with γ-irradiated *M. tuberculosis*, but this finding was not significant after controlling for foreign birth. There were no significant differences in IL-1β production 12 and 48 h after macrophage stimulation (data not shown).

**Table 3:** Median IL-1β production of macrophages assessed 24 h after two hours of stimulation

Factor-by-Group	Interleukin-1β production (in μg/mL)					
	Unadjusted difference in median expression			Adjusted difference in median expression^a^		
	β	95% CI	*P*-value	β	95% CI	*P*-value
LpqH						
Uninfected	Ref.	Ref.		Ref.	Ref.	
Latent TB infection	−11.95	(−33.51, 9.61)	0.27	−9.62	(−36.41, 17.16)	0.47
Pulmonary TB	−8.95	(−38.11, 20.21)	0.54	−8.95	(−44.03, 26.13)	0.61
Extrapulmonary TB	38.88	(8.45, 69.3)	0.01**	44.99	(−68.86, 158.86)	0.43
Gamma-irradiated *M. tuberculosis*						
Uninfected	Ref.	Ref.		Ref.	Ref.	
Latent TB infection	−44.28	(−88.91, 0.36)	0.05**	−37.92	(−86.66, 10.81)	0.12
Pulmonary TB	−12.89	(−71.47, 45.69)	0.66	− 12.89	(− 101.65, 75.87)	0.77
Extrapulmonary TB	−16.56	(−74.34, 41.23)	0.57	−16.07	(− 143.80, 111.66)	0.80
Live *M. tuberculosis*						
Uninfected	Ref.	Ref.		Ref.	Ref.	
Latent TB infection	−14.07	(−43.92, 15.79)	0.35	−14.07	(−116.22, 88.08)	0.78
Pulmonary TB	160.14	(−15.26, 335.53)	0.07	160.14	(−15.87, 336.14)	0.07
Extrapulmonary TB	16.71	(− 131.40, 164.81)	0.82	−4.05	(− 234.47, 226.38)	0.97

Unadjusted and adjusted quantile regression analysis of the four study groups according to stimulus used: LpqH, gamma-irradiated *M. tuberculosis*, and live *M. tuberculosis*

LpqH: 19 kDa lipoprotein of *M. tuberculosis*; Ref.: reference group; Family-wise error rate controlled for multiple comparisons using the Holm-Bonferroni method; ^a^Adjusted for foreign-born status

Holm-corrected *P*-values significant if < 0.0056; **Significant if using *P*-values uncorrected for multiple comparisons (*p* < 0.05)

To determine whether the ability of macrophages to kill *M. tuberculosis* in vitro differed by patient group, we performed in vitro killing assays of *M. tuberculosis*-infected macrophages that were incubated alone, with autologous PBMCs which were pre-incubated with γ-irradiated *M. tuberculosis*, or autologous PBMCs that were unstimulated. Overall, the CFU were lower in persons with previous extrapulmonary tuberculosis (indicating more robust killing of *M. tuberculosis),* but these findings were not statistically significant.

## Discussion

We found that *M. tuberculosis*-stimulated macrophages from persons with previous extrapulmonary TB had higher expression of VDR compared to macrophages from persons with previous pulmonary TB, TB contacts with latent *M. tuberculosis* infection, and uninfected contacts. This finding persisted after controlling for foreign birth, but not multiple testing. The latter was likely due to the small sample size. Median 25-hydoxyvitamin D levels were similarly low in all four patient groups, thus making the increased VDR expression in those with previous extrapulmonary TB notable. Given the large effect size of the difference in VDR expression, and the known importance of vitamin D in TB pathogenesis, the finding is likely biologically relevant. To our knowledge, ours is the first study to have evaluated the vitamin D pathway in persons who have recovered from TB.

The role of vitamin D in modulating the immune response is indicated by the activity of the VDR in activated human monocytes, the ability of the active form of vitamin D, 1,25(OH)_2_D_3_, to inhibit T cell proliferation, and the ability of pathogen-activated macrophages to produce 1,25(OH)_2_D_3._ [33–37] VDR is a member of the nuclear receptor of transcription factors and, upon activation by 1,25(OH)_2_D_3_, binds response elements on DNA, and heterodimerizes with the retinoic acid receptor (RXR), resulting in expression or repression of downstream genes [38]. One such downstream target encodes cathelicidin, a cationic antimicrobial peptide that causes direct lysis of mycobacteria through the permeabilization of cellular membranes. It also has chemotactic activity for neutrophils, monocytes, and some T cells [39]. Of note, 1,25(OH)_2_D_3_, together with IFN-γ, controls proliferation of *M. tuberculosis* by human monocytes [36]. A possible mechanism for this observation is that stimulation of TLR2 leads to upregulation of the VDR gene and the gene encoding the enzyme that converts vitamin D to its active form (CYP27B1) in monocytes [22].

Previous studies have found that polymorphisms in VDR have been associated with increased risk of TB, particularly extrapulmonary TB [14, 40]. Specifically, in a recent study among persons with spinal TB, the *ff* genotype of the VDR *Fok1* polymorphism correlated with lower VDR mRNA and protein levels in the intervertebral disk tissues, and the clinical severity of disease [41]. Our findings may differ because we assessed systematic expression of VDR rather than at the site of disease, all of our patient had recovered from TB, and we included persons with several forms of extrapulmonary TB, not just those with spinal TB. Other VDR polymorphisms that have been associated with TB meningitis include *Taq1* and *Apa1* [42, 43] A possible mechanism for these findings could be that single nucleotide polymorphisms (SNPs) in VDR genes, including *Fok1,* can modulate expression of vitamin D which may in turn have downstream immunologic effects [44].

In this study, there was no significant difference in 25-hydroxyvitamin D levels in participants with previous extrapulmonary TB and the three control groups, though median levels were low in all groups. In previous work, we demonstrated that 25-hydroxyvitamin D levels after recovery from TB were lower than in controls without TB disease, after controlling for important confounders—suggesting that low 25-hydroxyvitamin D levels could have been present prior to TB disease, and therefore contributed to TB risk [31]. However, there were no significant differences between those with previous extrapulmonary vs. pulmonary TB, [31] consistent with findings from other studies of persons with active TB disease [15, 45].

We found in the unadjusted analysis that macrophages from persons with previous extrapulmonary TB produced more IL-1β after stimulation with LpqH compared to the other study groups. The effect size was similarly large after controlling for foreign-born status, but it did not achieve statistical significance. IL-1β production has been increasingly recognized as a mechanism through which vitamin D acts to kill mycobacteria [46]. Liu, et al. reported that LpqH triggered IL-1β activity in vitro, which, in synergy with vitamin D activation, resulted in increased expression of antimicrobial genes such as defensin beta 4 and cathelicidin. [32] More recently, Thobackgale, et al reported that HIV- infected persons on antiretroviral therapy with recurrent TB disease had elevated production of IL-1β following monocyte stimulation with Bacillus Calmette-Guerin (BCG) compared to HIV infected persons without recurrent TB [47].

We have previously shown that persons with previous extrapulmonary TB appeared to have a subtle immune defect with decreased cytokine production and lower CD4 lymphocyte counts compared to persons with previous pulmonary TB, persons with LTBI and uninfected contacts [8–10]. We also have shown that persons with previous extrapulmonary TB have increased T cell activation and increased frequency of regulatory T cells compared to the other patient groups [11]. The increased expression of VDR and IL-1β in persons with previous extrapulmonary TB suggests that even after successful anti-mycobacterial therapy, their macrophages remain “primed” and demonstrate a brisk response after re-stimulation with *M. tuberculosis*. Our results may indicate “trained immunity” – epigenetic modification of monocytes as a result of prior exposure to *M. tuberculosis.* Previous studies have noted increased production of IL-1β and TNF-α after healthy volunteers underwent BCG vaccination [48].

Our findings differ from Liu, et al. [32] in that in our study increased VDR expression was seen after stimulation with live *M. tuberculosis* only, but not after stimulation with the 19 kDa LpqH protein, or killed *M. tuberculosis*. One main difference between these two studies is that we analyzed the immune response to *M. tuberculosis* in persons with previous TB disease and TB contacts. The response to live *M. tuberculosis* is likely more relevant to disease pathogenesis than the response to a single protein, or killed *M. tuberculosis*.

Our study had several limitations. First, it is unclear whether *M. tuberculosis-*specific immune responses in persons who have completed anti-TB therapy are similar to immune responses prior to their developing TB disease. However, our aim is to identify potential predisposing immune factors that lead to the development of TB which is not possible in the midst of cytokine fluctuations that occur during active disease. Second, different sites of extrapulmonary TB may have different pathophysiology. We included several different TB manifestations in the present study which potentially makes our findings more broadly applicable to all persons with extrapulmonary TB, although we are not able to draw conclusions about factors that may influence whether a person develops disease at a specific site. Also, we did not study gene expression or cytokine production at the site of disease, which could differ from peripheral blood immune responses. Finally, our inferences were limited by a relatively small sample, given that we also corrected significance level thresholds to account for a potential confounding variable (foreign birth) and multiple testing. The magnitude of the difference between groups observed for VDR (an adjusted difference in median expression of 29 RVU, comparing those with extrapulmonary TB to uninfected individuals) likely represents a substantive effect, despite its borderline statistical significance (after multiple-testing correction).

## Conclusion

We found that persons with previous extrapulmonary TB had increased macrophage expression of VDR following stimulation with live *M. tuberculosis*. Although it is unclear whether this finding predisposed to development of extrapulmonary TB or was a consequence of disease, study of the former possibility—as a marker of persons at increased risk of developing extrapulmonary TB—is warranted. If confirmed in other populations, studies of novel, logistically feasible methods to detect VDR expression and therefore those potentially at high risk of TB, are warranted. Discovery of factors that determine reactivation of disease and extrapulmonary dissemination will advance TB prevention efforts by identifying immune responses for boosting by TB vaccines. It will help identify those persons at increased risk for progression from latent infection to clinical disease for more intensive observation and treatment.

## Additional file

Additional file 1: **Table S1.** Taqman Gene Expression Assays used for qt-PCR. Table of Taqman primer probe sets for assayed genes. (DOCX 13 kb)

## Abbreviations

EPTB: Extrapulmonary Tuberculosis
H37Rv: virulent strain of *M. tuberculosis*
IGRA: Interferon gamma release assay
IL-1β: Interleukin 1 beta
LpqH: *M. tuberculosis* 19Kda lipoprotein
LTBI: latent TB infection
PBMC: Peripheral blood mononuclear cells
qPCR: quantitative PCR
TB: Tuberculosis
TLR2: Toll-like receptor 2
TNF-α: Tumor necrosis factor alpha
TST: Tuberculin skin test
VDR: Vitamin D receptor
