# Supplementary material for: Increased vitamin D receptor expression from macrophages after stimulation with M. tuberculosis among persons who have recovered from extrapulmonary tuberculosis
Source: BMC Infect Dis. 2019 Apr 30;19:366. doi: 10.1186/s12879-019-3958-7 (PMC6492421; doi:10.1186/s12879-019-3958-7)
Supplement: Supplementary file 1 — Table S1. Taqman Gene Expression Assays used for qt-PCR. Table of Taqman primer probe sets for assayed genes. (DOCX 13 kb) [file 12879_2019_3958_MOESM1_ESM.docx]

|  | | |
| --- | --- | --- |
| Gene Symbol | GenBank accession # | Assay ID |
| TNF-α | NM_000594.3 | Hs01113624_g1 |
| VDR | NM_000376 | Hs00172113_m1 |
| CATH | NM_004345 | Hs00189038_m1 |
| TLR2 | NM_001318787 | Hs01872448_s1 |
| GAPDH | NM_001289746 | Hs99999905_m1 |

**Supplementary Table S1**. Taqman Gene Expression Assays used for qt-PCR
